# Cell Nuclei Segmentation in Cytological Images Using Convolutional Neural Network and Seeded Watershed Algorithm

**DOI:** 10.1007/s10278-019-00200-8

**Published:** 2019-06-03

**Authors:** Marek Kowal, Michał Żejmo, Marcin Skobel, Józef Korbicz, Roman Monczak

**Affiliations:** 1 Institute of Control and Computation Engineering, University of Zielona Góra, Szafrana 2, 65-516 Zielona Góra, Poland; 2 Department of Pathology, University Hospital in Zielona Góra, Zyty 26, 65-046 Zielona Góra, Poland

**Keywords:** Convolutional neural networks, Watershed, Nuclei segmentation, Breast cancer, Oversegmentation, Mathematical morphology

## Abstract

Morphometric analysis of nuclei is crucial in cytological examinations. Unfortunately, nuclei segmentation presents many challenges because they usually create complex clusters in cytological samples. To deal with this problem, we are proposing an approach, which combines convolutional neural network and watershed transform to segment nuclei in cytological images of breast cancer. The method initially is preprocessing images using color deconvolution to highlight hematoxylin-stained objects (nuclei). Next, convolutional neural network is applied to perform semantic segmentation of preprocessed image. It finds nuclei areas, cytoplasm areas, edges of nuclei, and background. All connected components in the binary mask of nuclei are treated as potential nuclei. However, some objects actually are clusters of overlapping nuclei. They are detected by their outlying values of morphometric features. Then an attempt is made to separate them using the seeded watershed segmentation. If the attempt is successful, they are included in the nuclei set. The accuracy of this approach is evaluated with the help of referenced, manually segmented images. The degree of matching between reference nuclei and discovered objects is measured with the help of Jaccard distance and Hausdorff distance. As part of the study, we verified how the use of a convolutional neural network instead of the intensity thresholding to generate a topographical map for the watershed improves segmentation outcomes. Our results show that convolutional neural network outperforms Otsu thresholding and adaptive thresholding in most cases, especially in scenarios with many overlapping nuclei.

## Introduction

According to global cancer project (GLOBOCAN), breast cancer is the most common cancer among women worldwide. It was estimated that in 2012, nearly 1.7 million new cases were diagnosed (second most common cancer overall) and 521,907 cases of deaths due to breast cancer occurred (fifth cause of death from cancer overall). This represents about 12% of all new cancer cases and 25% of all cancers in women [3].

Breast cancer is mostly diagnosed by three medical examinations usually occurring in the following order: palpation, ultrasonography or mammography, and fine needle biopsy (FNB). In this work, we concentrate on the analysis of results of FNB examination in an automatic way. In FNB method, a cytological material is gathered directly from tumor using a fine needle. Next, it is fixed and stained with hematoxylin and eosin. Finally, glass slide with the cellular material is examined by the pathologist under the microscope. FNB is less traumatic and much safer for a patient than an open surgical biopsy. However, detection of cancer cells on the slide glass is not an easy task. Novice pathologists to gain experience and knowledge to become professional specialist must spend a lot of time browsing various cytological samples. Moreover, analysis of entire slide is a time-consuming process, even for experienced pathologists.

During the examination of cytological images, pathologists evaluate morphometric features of cells and their nuclei in order to distinguish tumor type. In recent years, we have seen a very intensive development of techniques dedicated to microscopic digital imaging. More and more specialists browse virtual slides on a computer screen instead of examining glass slides under a microscope. This situation opens up the possibility of supporting the pathologist’s work using modern image processing and machine learning techniques. They can speed up diagnosis and increase their accuracy. However, we must remember that the accuracy of nuclei segmentation is critical to the performance of computer-assisted cytology. Unfortunately, cytological images are rather challenging for existing segmentation methods because nuclei have the tendency to create complex structures like clumps or nests.

The most common approaches for nuclei segmentation are based on active contours, intensity thresholding, mathematical morphology, region growing, watershed, and deep learning [4, 6, 10, 11, 18]. Last years brought an enormous progress in classification and object recognition using Convolutional Neural Networks (CNN) [2, 29]. It seems to be a promising technique for semantic segmentation of cytological images [24, 25]. The most considerable advantage of CNN is its ability to classify a single pixel based on its neighborhood described by high-level features. That is why excellent results are obtained while detecting the nuclei and cytoplasm even when their staining is strongly heterogeneous. Problems arise when nuclei overlap and there are no clear boundaries between them. In this situation, ordinary CNN networks are not able to separate such nuclei. It seems that watershed method deals better with such cases. However, the results obtained by the watershed are strongly dependent on the quality of the topographical map of distances. To obtain precise topographical map, we need a precise mask of nuclei region. Usually, intensity thresholding or extended h-minima are used for this purpose [7, 23]. Unfortunately, intensity thresholding usually generates nuclei mask that contain objects with jagged contours and clumped objects. As an effect, watershed usually is affected by the over-segmentation. On the other hand, the results of extended h-minima can vary substantially with regard to the value of chosen *h*. According to the results presented in [16], the method can miss finding some nuclei for some values of *h*.

To tackle these problems, we propose an approach that combines the advantages of CNN and the watershed method. CNN is employed to detect precise nuclei mask, which then is used to generate topographical map and nuclei seeds for watershed. Watershed is applied to separate overlapping nuclei. Experimental studies have shown that the use of CNN instead of the usual thresholding to determine the nuclei mask significantly increases the accuracy of nuclei segmentation.

The remainder of this paper is organized as follows. Section “Data” presents material used for experiments. The details of the proposed approach are described in the “Methods” and “Data Preprocessing”sections. Section “Experiment” presents the details of the experiments carried out. Their results are shown in the “Results” section. The paper ends with conclusions and future research in the “Conclusions and Further Research” section.

## Data

Cytological images used in this study came from 40 patients of the University Hospital in Zielona Góra, Poland. Half of the cases are malignant, half are benign. All tumors were histologically confirmed, and all patients who had a benign disease were biopsied or followed up for a year. Cellular material was acquired from affected tissue using 0.5-mm-diameter needle under the control of an ultrasonograph. The material was fixed and then dyed with hematoxylin and eosin (H+E). Glass slides were scanned using VS120 Olympus Virtual Microscopy System. The system consists of a 40x lens and 2/3” CCD camera giving 0.17 *μ* m resolution. As a result, 40 slides were generated. The average size of slide is approximately 200*k* × 100*k* pixels. For each virtual slide 2 regions of interests (ROI) of size, 1583 × 828 pixels representing malignant or benign cells were selected and saved as 8 bit/channel RGB TIFF image. Example of ROI selected by the pathologist from the virtual slide is shown in Fig. 4.

In total, we have collected 80 images. We divided them into subset 1 (40 images coming from 10 benign cases and 10 malignant cases) and subset 2 (40 images coming from the rest 10 benign cases and the rest 10 malignant cases). Subset 1 was used to train and validate CNN, subset 2 was used as a test subset to verify the accuracy of the proposed approach. To train CNN, we have divided subset 1 (40 images) into training subset (20 images coming from 5 malignant cases and 5 benign cases) and validation subset (20 images coming from 5 malignant cases and 5 benign cases). Thus, images used for training CNN were never used to the validation procedure. Moreover, the experiment conducted to verify the effectiveness of the segmentation procedure was carried out using test images. They do not include images coming from patients that were used for training and validation of CNN.

## Methods

In this section, we describe the main steps of the proposed segmentation method: (1) semantic segmentation of nuclei and background using CNN; (2) determining connected object on the semantic map generated by CNN; (3) detection of connected clusters of objects (clustered nuclei) based on their area and roundness; (4) applying conditional erosion to determine nuclei seeds among clumped objects; (5) separation overlapping nuclei using seeded watershed; (6) aggregating segmentation results for overlapping and non-overlapping nuclei. The first step is implemented using Python and Keras library, the rest of the steps are implemented using Matlab.

The watershed transform is one of the most often used segmentation method to separate touching or overlapping objects. However, the method is effective if proper seeds of objects are given. Here, we are proposing conditional erosion to detect centers (seeds) of prospective nuclei. Erosion is carried out on nuclei mask. Therefore, it is crucial for the method to determine precise mask of the nuclei. If nuclei overlap, we need at least to see some parts of their silhouettes to determine their centers using conditional erosion. Overlapping nuclei on inaccurate nuclei mask usually create huge clumps and thus even conditional erosion will fail to find their centers. To deal with this challenge, we propose CNN as a tool for recognizing nuclei regions. We also used to segment nuclei regions two other methods based on intensity thresholding: Otsu thresholding (GO) and adaptive thresholding (AT). Finally, we compared the accuracy of CNN with both intensity thresholding approaches.

### Convolutional Neural Network

In recent years, CNN has gained a lot of popularity as a tool for image segmentation and object recognition [12]. Typical CNN is usually comprised of at least with two convolutional layers combined with pooling layers and ended by at least one fully connected layer (Fig. 6).

**a) Convolutional layer**: is a core part of CNN, composed of a set of learnable filters. Each filter extracts different features from the input image. Filter parameters (weights) are tuned during the learning procedure.
**b) Pooling layer**: is used to progressively reduce the spatial size of the input in order to extract higher level features. Spatial size reduction is usually done by max pooling using window of size 2×2 pixels.
**c) Fully connected layer**: is at the end of CNN and is connected to all activations in the previous layer. The input of this layer is a one-dimensional feature vector. The task of this layer is to capture the complex relationships between high-level features and output labels.

Trained CNN model was used for semantic segmentation. It classifies each pixel from the input image into one of four categories (nuclei, cytoplasm, nuclei edge, background). In fact, the output of CNN contains probability distribution over four classes. Therefore, each pixel is always labeled by the class which gained the highest probability. As a result, we get a semantic mask for input image.

### Segmentation of Nuclei Region

**Fig. 1:**
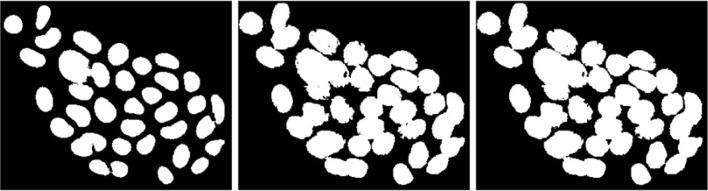
Segmentation of nuclei region: CNN (left), AT (middle), and GO (right)

In this step, semantic mask generated by CNN model is transformed into nuclei mask. Pixels belonging to nuclei are labeled by 1, while others by 0. Therefore, such mask is very similar to binary masks generated by GO or AT method. Figure 1 presents sample results for CNN, GO, and AT. We can visually asses that CNN is much more precise in nuclei segmentation than GO and AT. It can be observed that CNN separates the nuclei that are touching and overlapping much better than two other techniques.

### Detection of Overlapping Nuclei

**Fig. 2:**
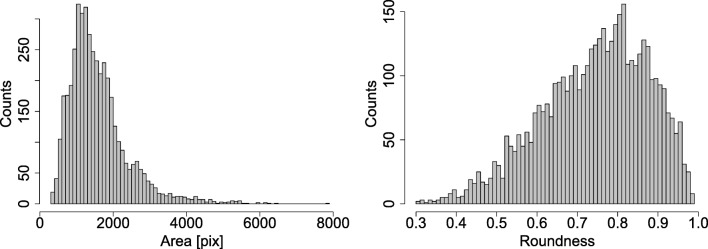
Histograms of the nuclei areas and roundness

All cytological images used in this study were manually annotated by marking nuclei contours. Therefore, it is possible to compute morphometric features of nuclei and determine their distributions (see Fig. 2). We decided to describe nuclei by their area and roundness [20]:

1 $$ Roundness = \frac{4 \times Area}{{(Perimeter)}^{2}}.  $$

Based on 4447 manually annotated nuclei, it can be concluded that nuclei have area in the range from 309 to 7801 pixels and roundness in the range from 0.31 to 0.99. Based on these findings, we are able to distinguish the nucleus from the object which consists of many clumped nuclei.

Nuclei mask determined by CNN usually contain two types of regions that we call non-overlapping or overlapping. In non-overlapping regions, objects are easily recognized as nuclei because they are well separated, and thus their areas and roundness are within allowed limits. These objects are immediately classified as nuclei and they do not require further processing. The rest of the objects that do not meet the requirements of being single nucleus must be subjected to a separation procedure by seeded watershed.

### Separating Overlapping Nuclei Using Seeded Watershed

The classical watershed transform treats the image to be segmented as a topographic surface. It segments image by flooding basins from the seeds until basins attributed to different seeds meet on watershed lines. The input of the algorithm is usually a binary mask of the image. It is transformed by the Euclidean distance transform and local maxima from this transform are used as seeds. Unfortunately, algorithm in this form tends to create many micro-segments. Such over-segmentation makes the results of the watershed method completely useless. To deal with this problem, we used extended version of watershed that uses nuclei seeds generated by the conditional erosion [9, 30]. The method process binary mask to detect centers of objects. In our case, it was applied to nuclei mask *I*_*NM*_ to find nuclei centers *I*_*S*_ (seeds). We applied this processing only to the objects classified as nuclei clumps.

In our approach, conditional erosion is based on classical erosion defined as the operation of structuring element *B* on image *I*_*NM*_:

2 $$ I_{NM} \ominus \check{B} = \{x\in \mathbb{R} | (B+x) \subset{I_{NM}}\}, $$

where $\check {B}$ is a reflection of set *B*. Conditional erosion is conducted in two steps. First, coarse erosion using structuring element *M*_*c*_ reduces the size of objects. To prevent objects from disappearing, coarse phase switches to fine phase for objects with area below *T*_1_. Fine erosion uses structuring element *M*_*f*_ which is less likely to make the nucleus disappear. It tries to separate clustered nuclei. Element *M*_*f*_ is used iteratively until all objects have area below *T*_2_. The size of both structuring elements should be significantly smaller than the size of the processed objects to not to reduce them too rapidly. Based on the reference nuclei (manually segmented), we know that the size of nuclei can vary from 300 to 8000 pixels, thus we can use the coarse structuring element *M*_*c*_ and fine structuring element *M*_*f*_ proposed in [30]:

$$\scriptsize M_{c}=\left( \begin{array}{ccccccc} 0 & 0 & 0 & 1 & 0 & 0 & 0\\ 0 & 0 & 1 & 1 & 1 & 0 & 0\\ 0 & 1 & 1 & 1 & 1 & 1 & 0\\ 0 & 1 & 1 & 1 & 1 & 1 & 0\\ 0 & 1 & 1 & 1 & 1 & 1 & 0\\ 0 & 0 & 1 & 1 & 1 & 0 & 0\\ 0 & 0 & 0 & 1 & 0 & 0 & 0 \end{array}\right), \mkern30mu M_{f}=\left( \begin{array}{ccc}0 & 1 & 0\\ 1 & 1 & 1\\ 0 & 1 & 0 \end{array}\right). $$

To determine thresholds *T*_1_ and *T*_2_, we conducted a segmentation test on a few chosen images. Segmentation results were compared with those obtained by using classical watershed segmentation. We determined experimentally that the best segmentation is obtained for thresholds *T*_1_ = 350 and *T*_2_ = 50.

The prototype of topographic map *I*_*TM*_ is determined by Euclidean distance transform. Next, seeds *I*_*S*_ are used to refine the topographic map. Seeds *I*_*S*_ are combined with the original topographic map *I*_*TM*_ by morphological reconstruction $\rho _{I_{TM}}(I_{S})$ [28]. The algorithm is based on repeated dilations of a seed mask *I*_*S*_ until the contour of the seed mask fits under a topographic map *I*_*TM*_:

3 $$ I^{\prime}_{TM} = \rho_{I_{TM}}(I_{S}) = \bigcup\limits_{n \ge 1}\delta_{I_{TM}}^{(n)}(I_{S}). $$

**Fig. 3:**
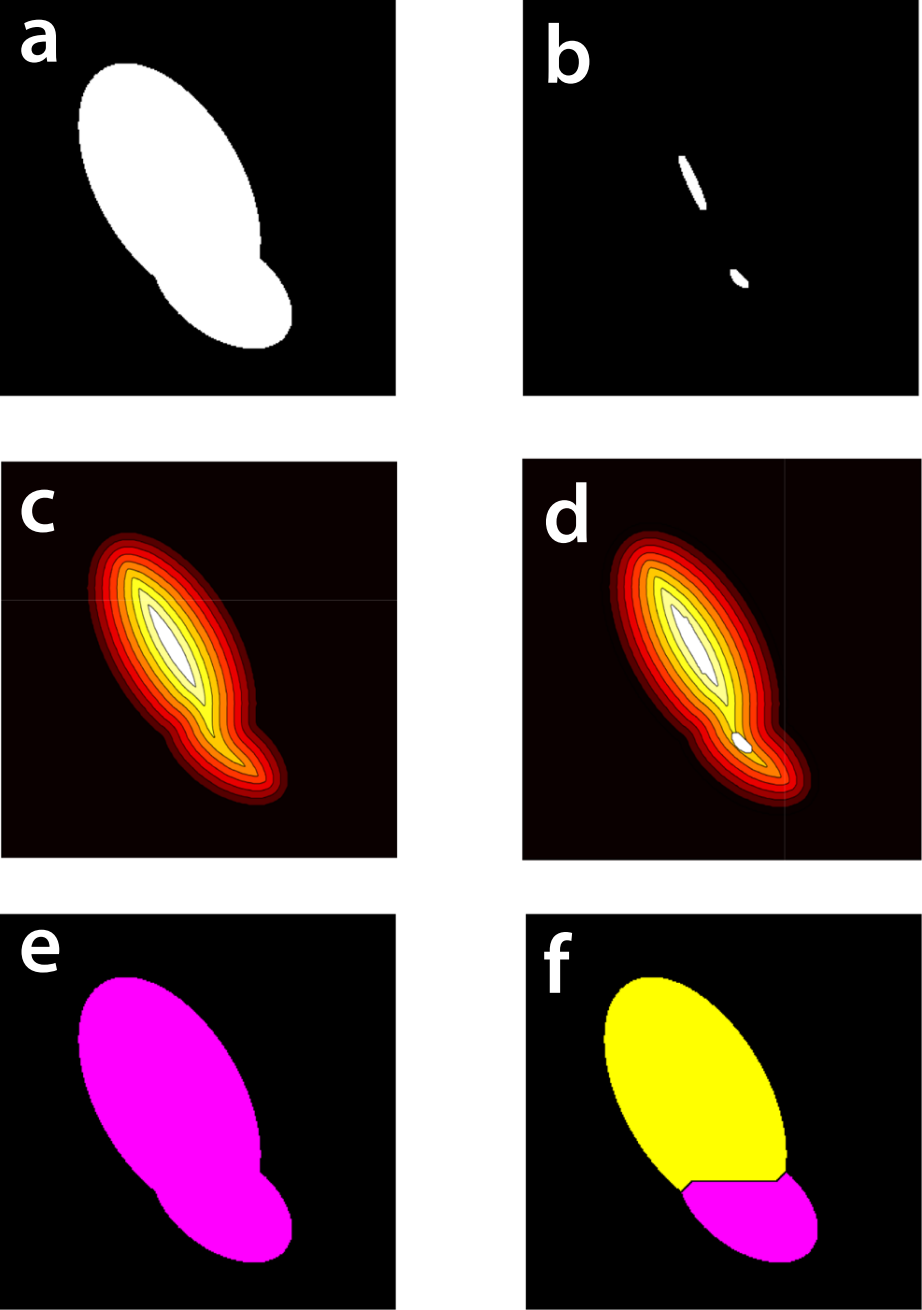
Segmentation of overlapping ellipses using seeded watershed and classical watershed: **a** input mask; **b** result of conditional erosion; **c** distance transform; **d** modified distance transform with imposed seeds; **e** watershed using distance transform; **f** watershed using seeded distance transform

The grayscale geodesic dilation of size *n* is then given by:

4 $$ \delta_{I_{TM}}^{(n)}(I_{S}) = \underbrace{\delta_{I_{TM}}({\ldots} \delta_{I_{TM}}(\delta_{I_{TM}}(I_{S})))}_{n}, $$

and the elementary geodesic dilation is described by the following relationship:

5 $$ \delta_{I_{TM}}(I_{S}) = (I_{S} \oplus B) \cap I_{TM}, $$

where (*I*_*S*_ ⊕ *B*) is a standard dilation of size one followed by an intersection (pointwise minimum ∩) and *B* is 4-connected neighborhood structural element with pair of horizontal and vertical connected pixels [28]. In Fig. 3, we can observe how the seeds generated by conditional erosion modify original topographic map and the positive effect of such approach on the segmentation of overlapping objects with the elliptic shape.

The approach described above is used to separate nuclei clusters found in nuclei mask by procedure described in the “Detection of Overlapping Nuclei” section. The whole separating procedure employing conditional erosion and watershed is applied to each object classified as overlapping objects (clumped nuclei). Therefore, we obtain separate segmentation results for each nuclei cluster. After the separation procedure by seeded watershed, objects detected are again classified as overlapping or non-overlapping nuclei using the method described in the “Detection of Overlapping Nuclei” section. We do this to check if the separation was successful. All nuclei that successfully pass the test of being single nucleus are included in the final segmentation results. Clusters of nuclei that have failed to be separated are rejected and do not participate in further processing.

## Data Preprocessing

Input images are preprocessed by color separation procedure and cutting into blocks of fixed size (patches). Patches are further processed and augmented to prepare training data for CNN.

### Color Separation

Cytological samples are subjected to a staining process to precisely visualize the cellular material that is being analyzed. We usually use hematoxylin and eosin for this purpose. Hematoxylin is mainly absorbed by the cell nuclei and dyes them blue. Eosin dyes the cellular material in red and deposits mainly in the cytoplasm. Unfortunately, the process of staining and digitizing glass slides is not standardized. In effect, cytological samples coming from different laboratories may differ in color. Color variation can arise due to different staining protocols, different stain brands, a shelf life of stains, or due to using different microscopy scanners. It has been shown that the performance of segmentation algorithms deteriorates substantially when the color of processed images differs from the color of training images [8, 15, 17]. To tackle this problem, various color normalization methods have been proposed. They can be generally categorized into histogram matching methods, color transfer methods, and spectral matching methods (For a complete overview of the state of the art color normalization methods please see [22]).

Color normalization is usually preceded by the stain separation because different cellular structures absorb all stains to some extent. The stain concentration is closely related to the attenuation of the light transmitted through the stained material. In turn, light transmission through the cytological sample can be described by the Beer Lambert law:

6 $$ I = I_{0}\exp{(-WH)}, $$

where *I* is the intensity of the light that passed the sample (3 × *n* matrix, *n* - number of pixels), *I*_0_ is the intensity of light entering the sample (matrix of the same size as *I*), *W* is a stain color matrix of size 3 × *k* (*k* = number of stains), and matrix *H* (*k* × *n*) represents the stain concentrations. Stain separation approaches can be divided into supervised and unsupervised methods. The well-known supervised method is based on color deconvolution [21]. In this method matrix, *W* is determined empirically and it uses pseudo inverse transform to obtain *H*. By contrast, unsupervised methods estimate both *W* and *H*. Usually these methods are based on independent component analysis (ICA) or non-negative matrix factorization (NMF) [1, 19]. Another approach is based on a model where image colors are linear combinations of stain vectors which describe the proportion of absorbed light in RGB channels. Due to the fact that absorption weights are non-negative, thus every value may exist between stain vectors. The method exploits this fact to find them using two largest singular values determined by singular value decomposition (SVD) [14].

**Fig. 4:**
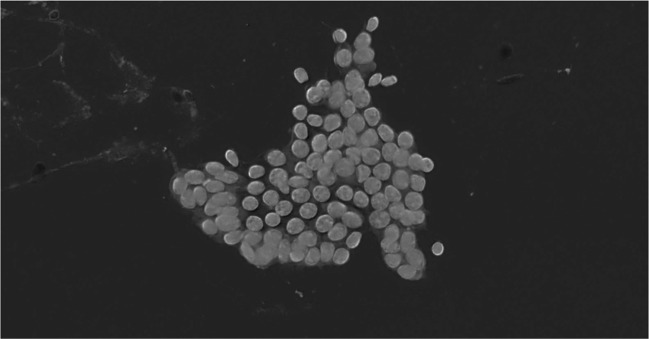
Image representing hematoxylin concentration

In this study, we applied a supervised method of stain separation proposed in [21]. The effectiveness of this algorithm is confirmed by numerous research publications and moreover it is easily available for many scientific computing environments. Our implementation is based on built-in stain vectors for Hematoxylin/Eosin taken from ImageJ color deconvolution plugin. Absorption spectra of hematoxylin and eosin overlap in RGB space, but mentioned color separation allows us to some extent evaluate the contribution of hematoxylin and eosin at each pixel. Three separate intensity images are created as a result of deconvolution, the first represents the hematoxylin concentration, second eosin concentration, and third residuals. For further processing, we are using images of hematoxylin concentration. They emphasize nuclei and suppress cytoplasm which absorbs mainly eosin (Fig. 4).

After color separation, hematoxylin image is subjected to feature-wise standardization. Each pixel in the image array is treated as a separate feature. Based on the randomly chosen sample of 10,000 training image patches, we can determine the mean and standard deviation for every feature (pixel). They are stored and then used to standardize images. Every pixel in the image is standardized using a mean and standard deviation determined exactly for his position in the image

### Manual Segmentation

To train CNN, we needed ground truth images with labeled objects. Therefore, all images used in this study were subjected to manual segmentation. The procedure was carried out using ImageJ software^1^ and involved selection of four types of objects: nuclei interior, nuclei contour, cytoplasm, and background. In the case of ambiguity, e.g., if overlapping nuclei cannot be separated, no objects were marked. But, if the separation was possible, then nuclei were marked as separate objects.

Based on selected regions, semantic maps were created for each image. On a semantic map, each pixel is given the label and can belong to the following classes: indeterminate, background, cytoplasm, nuclei, or nuclei border. Set of pixels belonging to nuclei border was determined automatically by extracting pixels from nuclei regions lying on the contours of nuclei.

### Extraction of Patches

**Fig. 5:**
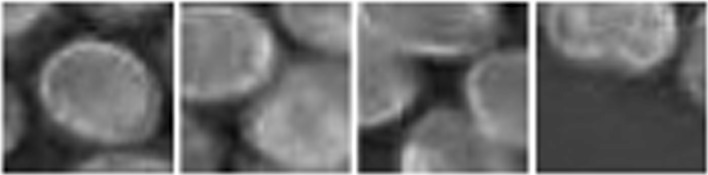
Example of training patches (hematoxylin concentration). From left: nuclei, nuclei border, cytoplasm, background

The images were cut into patches of size 43×43 pixels. The size of the patch was chosen arbitrary so that the patches contain large fragments of cell nuclei. For each pixel, a single patch is extracted. The class of each patch is assigned based on the label of central pixel. Label is read from semantic map. Patches corresponding to unlabelled pixels are excluded from further processing and thus are not used to train or validate CNN. As in many other studies concerning semantic segmentation, CNN is deciding about the class of the pixel based on the patch centered in that pixel. Set of all patches was divided into training and validation subsets, their sizes, and distributions are presented in Table 1. Examples of patches used for training CNN are shown in Fig. 5.

**Table 1:** Collections of patches

	Training	Validation
Number of images	20	20
Total patches	18,315,912	18,961,569
− nuclei border	1,739,200	1,968,232
− nuclei center	5,105,059	5,788,305
− cytoplasm	5,588,398	5,770,039
− background	5,883,255	5,434,993

### Patches Augmentation, Transformation, and Preprocessing

The raw patches generated in the previous step were augmented and preprocessed to improve the learning process of the CNN model. It is important for learning procedure to have balanced number of training samples within each class. In our case, the class of patches describing nuclei borders is underrepresented (see Table 1). To overcome this problem, a set of nuclei border patches was augmented by artificial patches. They were generated using some well-known transformations applied to actual patches. To generate new patches, each original nuclei border patch was subjected to three randomized transformations: scaling by a factor from range 0.8 to 1.2, rotating using random angle, flipping vertically and/or horizontally. The set of patches was enlarged four times using augmentation technique. To increase the diversity and variability of patches coming form other classes, they were also randomly subjected to these transformations. In order not to increase the size of these classes, the original patch after being transformed was replaced by the new one.

## Experiment

### Network Architectures and Training Parameters

**Fig. 6:**
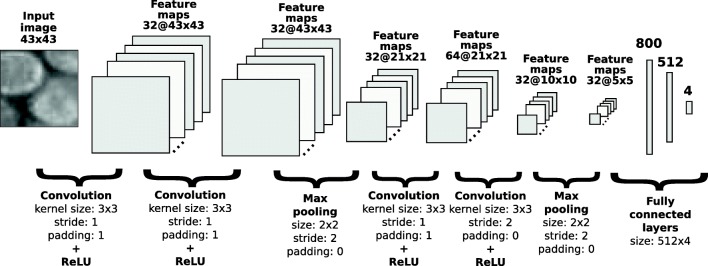
The structure of CNN

A lot of different CNN architectures have been already described and tested in the scientific literature [12, 13, 27]. They vary in the layer configuration and depth of the structure. The structure of CNN used in this study is shown in Fig. 6. Our network consists of four convolutional layers, which are separated by two max-pooling layers. At the top of the network, we placed two fully connected layers (512 neurons and 4 neurons respectively). All convolutional layers are followed by rectified linear units (ReLU). Weights in convolutional layers were initialized using Xavier method, learning rate was set to 10^− 4^, and weight decay was set to 10^− 6^ [5]. Training was conducted using stochastic gradient descent, mini-batch was set to 256, and training process was finished after 20 epochs. We applied dropout technique to prevent the network from over-fitting [26]. This allowed us to achieve the 91.33% classification accuracy for the validation set. All experiments were realized with the help of GeForce GTX TITAN X with 12GB of RAM.

### Semantic Segmentation

**Fig. 7:**
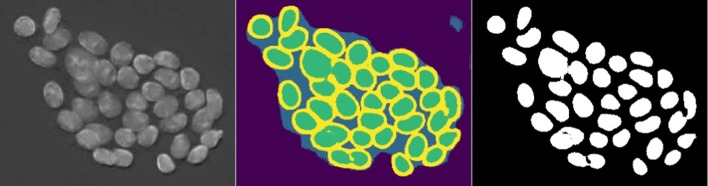
Results of semantic segmentation using CNN: input image: hematoxylin concentration (left), semantic mask generated by CNN classifier (middle), and nuclei mask (right)

Trained CNN model was used to predict classes for all pixels in the images from the test set. The input of the network is a single patch. The output is a class probability distribution for a central point of the patch. To segment the whole image, classification procedure must be repeated for each pixel in the image. As a result, we obtain a class probability distribution for every pixel. Finally, pixel is labeled by the class that achieved the highest probability. In this way, a semantic mask was obtained for a given input image. From the semantic mask, it is possible to extract nuclei mask to generate a topographic surface for the watershed transform. Sample semantic mask and nuclei mask are shown in Fig. 7.

## Results

### Evaluation Procedure

In order to verify the effectiveness of the proposed approach, it was applied to detect nuclei in 20 test images. The accuracy of CNN was compared with the accuracy of GO and AT method. These methods were used to determine the nuclei mask but final segmentation was always carried out using seeded watershed (see “Segmentation of Nuclei Region”). Thus, three methods used to extract nuclei mask were compared with respect to watershed segmentation accuracy.

To measure the accuracy of automatic segmentation, we compare nuclei segmented automatically with reference nuclei segmented manually. We are given a list of manually segmented nuclei in the form of binary masks. Nuclei segmented automatically can be also presented in the form of binary masks. Therefore, it is possible to measure distances between reference nuclei and automatically segmented nuclei. To do that, we are using Hausdorff distance (HD) and Jaccard distance (JD). For each segmentation method, (CNN + seeded watershed, GO + seeded watershed, AT + seeded watershed), two distance matrices were determined using Hausdorff distance and Jaccard distance respectively. The distance matrix stores the distances between all pairs of manually segmented nuclei and nuclei segmented using a chosen automatic segmentation method. Using distance matrices, we are trying to pair all manually segmented nuclei with the closest nuclei segmented automatically. We assumed that the nuclei between which Hausdorff distance is greater than 30 or Jaccard distance is greater than 0.5 are so different that must be considered as two separate objects. The single manually segmented nucleus can be paired with the only one, nearest nucleus segmented automatically and the distance between them must be below the predefined threshold. As a result, 3 scenarios are possible: manually segmented nucleus can be matched with the nearest automatically segmented nucleus and such case is classified as true positive (TP), no automatically segmented nucleus can be found to match with the manually segmented nucleus and such case is classified as false negative (FN), and automatically segmented nucleus can stay without corresponding manually segmented nucleus and thus it is classified as false positive (FP).

### Experimental Results

**Fig. 8:**
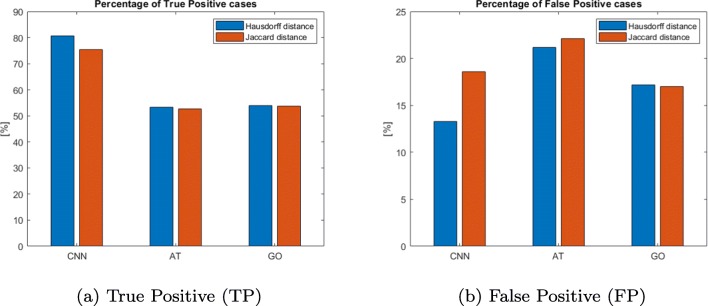
Aggregated TP rates and FP rates with regard to methods of segmentation

Tables 2 and 3 summarize basic statistics for TP and FP coefficients determined for all test images. The results are presented separately for benign and malignant cases. Aggregated (for benign and malignant cases) mean values of TP and FP rates are shown in Fig. 8.

**Table 2:** Results for Hausdorff distance

	TP			FP		
	CNN	AT	GO	CNN	AT	GO
Benign						
Mean	*83.4%*	52.4%	51.3%	*5.1%*	14.6%	14.9%
Sd	*12.8%*	21.6%	19.0%	*4.1%*	5.0%	5.8%
Max	*98.2%*	86.1%	77.2%	*15.5%*	23.1%	30.8%
Min	*41.0%*	7.7%	10.3%	*0.0%*	3.8%	6.3%
Malignant						
Mean	*78.1%*	54.4%	56.6%	21.4%	27.9%	*19.2%*
Sd	11.7%	*10.8%*	12.1%	13.4%	12.4%	*7.2%*
Max	*93.7%*	77.8%	85.2%	51.9%	63.6%	*32.7%*
Min	*56.3%*	38.7%	37.0%	*3.2%*	14.5%	8.1%

The best results are italicized

**Table 3:** Results for Jaccard distance

	TP			FP		
	CNN	AT	GO	CNN	AT	GO
Benign						
Mean	*77.6%*	50.1%	49.6%	*10.9%*	16.9%	16.5%
Sd	*14.2%*	22.6%	20.0%	*6.5%*	*6.5%*	7.6%
Max	*93.3%*	81.9%	76.8%	*24.6%*	27.4%	38.5%
Min	*38.5%*	7.7%	10.3%	*1.3%*	7.1%	7.5%
Malignant						
Mean	*73.2%*	55.1%	57.8%	26.3%	27.2%	*18.0%*
Sd	11.4%	*10.9%*	13.7%	11.2%	11.9%	*6.7%*
Max	90.5%	81.5%	*92.6%*	52.7%	54.5%	*29.3%*
Min	*53.1%*	38.7%	37.5%	12.0%	10.9%	*3.7%*

The best results are italicized

We can observe that CNN method outperforms GO and AT for benign cases. On average, CNN-based approach can extract 83.4% of benign nuclei according to HD and 77.6% according to JD. Whereas the methods based on GO and AT are able to extract from 51.3 to 52.4% of benign nuclei according to HD and from 49.6 to 50.1% according to JD. We also see that CNN-based segmentation outperforms other methods for malignant cases. For malignant cases, CNN-based method can correctly detect 78.1% of nuclei according to HD and 73.2% according to JD, while for the other methods average values are in range 54.4%–56.6% for HD and 51.1%–57.8% for JD.

The CNN method received on average lower FP error for benign cases than the other methods, while for malignant cases FP error is slightly larger than for GO method. The obtained values of standard deviations indicate that for all methods results are distributed in similar range around the mean.

**Fig. 9:**
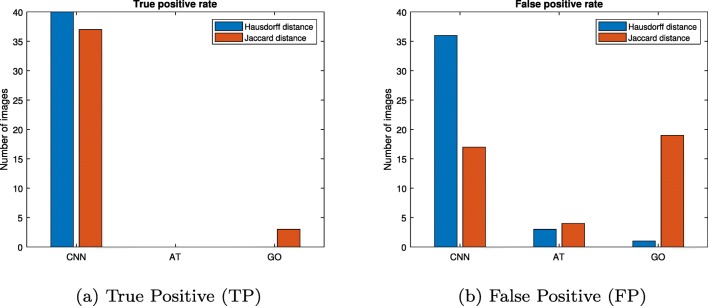
The number of images for which the segmentation method obtained the best result

In Fig. 9, we have presented results that indicate how many times each segmentation method reached the highest TP rate and the lowest FP rate. CNN clearly outperforms two other approaches, because it gained the best results for most test images.

**Fig. 10:**
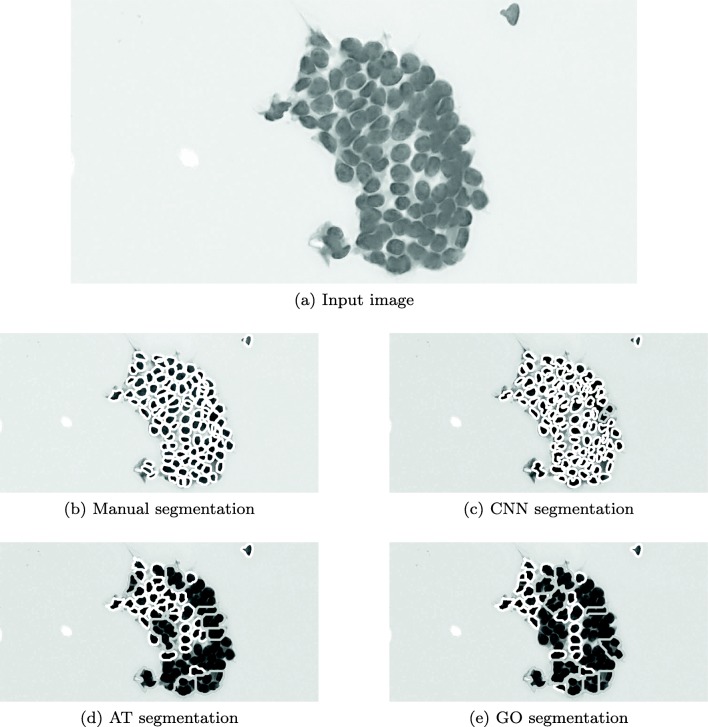
Sample segmentation results: TP (white), FP (gray)

Classical intensity thresholding methods (AT, GO) are very effective for detecting areas occupied by the nuclei if nuclei are well separated. Unfortunately, these methods usually fail if overlapping nuclei are present in an image. This problem is much less visible if we use CNN instead of GO or AT. Sample segmentation results confirming this fact are shown in Fig. 10. The CNN method is more effective than AT and GO in detecting overlapping objects, but the side effect is the detection of redundant objects. Thus, the value of FP for some images can slightly increase if we use CNN segmentation. However, on average CNN method performs much better than GO and AT.

## Conclusions and Further Research

Methods of nuclei segmentation based on intensity thresholding, edge detection, watershed transform, active contours and artificial neural networks usually have problems with cytological images. Mainly because of complex and heterogeneous nature of these images which often contain overlapping and touching nuclei. In this paper, we are presenting an alternative approach which uses CNN classifier to pre-segment nuclei and then seeded watershed to deal with overlapping nuclei. We are showing that our approach outperforms classical watershed method. Moreover, we determined that the proposed method can detect about 81% of real nuclei (on average) and, at the same time, have a low rate of false positive detection (13%).

Despite the fact that obtained result looks quite promising, there are some points of our project which can be improved. Semantic segmentation using a classical convolutional neural network turned out to be very computationally expensive. Especially training process was time consuming. In future research, we are going to use for semantic segmentation fully convolutional network (e.g., U-Net network) which will allow us to significantly reduce the computational burden of the method.

Another drawback of the method is that it needs manually segmented nuclei to train CNN. Unfortunately, the process of manual segmentation is very time consuming and tedious. Therefore, we plan to develop a software for semi-automatic segmentation of nuclei, an operator will have to only correct the errors introduced by the automatic method. Such approach allows us to prepare richer set of training images and save time.

We also plan to modify the step responsible for detection of overlapping nuclei coming from semantic segmentation. We are going formally determine the thresholds for roundness and area based on hypothesis testing framework.

Our system can be used to segment and detect nuclei. Therefore, we plan to develop a computer-aided cytology system to help diagnose breast cancer and lung cancer. Based on the segmentation results of our system, we plan to compute morphometric, textural, and colorimetric features of nuclei and apply machine learning techniques to make predictions about the type of the cancer.

## Footnotes

1 https://imagej.nih.gov/ij/

## References

[1] Alsubaie N, Trahearn N, Raza S, Snead D, Rajpoot N (2017). Stain deconvolution using statistical analysis of multi-resolution stain colour representation. PLoS ONE.

[2] Feng Z, Yang Z, Jin L, Huang S, Sun J (2017). Robust shared feature learning for script and handwritten/machine-printed identification. Pattern Recogn Lett.

[3] Ferlay J, Soerjomataram I, Dikshit R, Eser S, Mathers C, Rebelo M, Parkin DM, Forman D, Bray F (2013) Globocan 2012 v1.0, cancer incidence and mortality worldwide: Iarc cancerbase no. 11. http://globocan.iarc.fr 10.1002/ijc.29210 25220842

[4] Gertych A, Ma Z, Tajbakhsh J, Velasquez-Vacca A, Knudsen B (2016). Rapid 3-D delineation of cell nuclei for high-content screening platforms. Comput Biol Med.

[5] Glorot X, Bengio Y: Understanding the difficulty of training deep feedforward neural networks. In: (Teh YW, Titterington M, Eds.), vol 9. Sardinia: PMLR, 2010, pp 249–256

[6] Irshad H, Veillard A, Roux L, Racoceanu D (2014). Methods for nuclei detection, segmentation, and classification in digital histopathology: a review–current status and future potential. IEEE Rev Biomed Eng.

[7] Jung C, Kim C (2010). Segmenting clustered nuclei using H-minima transform-based marker extraction and contour parameterization. IEEE Trans Biomed Eng.

[8] Khan AM, Rajpoot N, Treanor D, Magee D (2014). A nonlinear mapping approach to stain normalization in digital histopathology images using image-specific color deconvolution. IEEE Trans Biomed Eng.

[9] Kowal M: Computer-aided diagnosis for breast tumor classification using microscopic images of fine needle biopsy. In: (Korbicz J, Kowal M, Eds.) Intelligent systems in technical and medical diagnostics, advances in intelligent systems computing: 230. Springer, Berlin, 2013, pp 213–224

[10] Kowal M, Skobel M, Nowicki N (2018). The feature selection problem in computer-assisted cytology. Int J Appl Math Comput Sci.

[11] Koyuncu C, Akhan E, Ersahin T, Cetin-Atalay R, Gunduz-Demir C (2016). Iterative h-minima-based marker-controlled watershed for cell nucleus segmentation. Cytom A.

[12] Krizhevsky A, Sutskever I, Hinton GE: Imagenet classification with deep convolutional neural networks.. In: Proceedings of 25th international Conference Neural information processing systems - volume 1, NIPS’12. Curran Associates Inc., Lake Tahoe, 2012, pp 1097–1105

[13] LeCun Y, Huang FJ, Bottou L: Learning methods for generic object recognition with invariance to pose and lighting.. In: Proceedings of IEEE computer society conference on computer vision and pattern recognition, CVPR, vol 2, Washington, 2004, pp 97–104

[14] Macenko M, Niethammer M, Marron JS, Borland D, Woosley JT, Guan X, Schmitt C, Thomas NE: A method for normalizing histology slides for quantitative analysis.. In: IEEE International Symposium Biomedical imaging: From nano to macro (ISBI), Boston, 2009, pp 1107–1110

[15] Nurzyńska K: Optimal parameter search for colour normalization aiding cell nuclei segmentation. In: (Kozielski S, Mrozek D, Kasprowski P, Malysiak-Mrozek B, Kostrzewa D, Eds.) Beyond databases, architectures and structures. Facing the challenges of data proliferation and growing variety, communications in computer and information science. Springer International Publishing, Berlin, 2018, pp 349–360

[16] Nurzyńska K, Mikhalkin A, Piórkowski A (2017). Cas: Cell annotation software - research on neuronal tissue has never been so transparent. Neuroinformatics.

[17] Peter L, Mateus D, Chatelain P, Schworm N, Stangl S, Multhoff G, Navab N: Leveraging random forests for interactive exploration of large histological images. In: (Golland P, Hata N, Barillot C, Hornegger J, Howe R, Eds.) Proceedings of Medical image computing and computer-assisted intervention – MICCAI 2014. Springer International Publishing, Boston, 2014, pp 1–8 10.1007/978-3-319-10404-1_1 25333094

[18] Piórkowski A, Nurzyńska K, Gronkowska-Serafin J, Selig B, Boldak C, Reska D (2017). Influence of applied corneal endothelium image segmentation techniques on the clinical parameters. Comput Med Imaging Graph.

[19] Rabinovich A, Agarwal S, Laris C, Price J, Belongie S: Unsupervised color decomposition of histologically stained tissue samples. In: (Thrun S, Saul LK, Scholkopf B, Eds.) Advances in Neural Information Processing Systems 16. MIT Press, 2004, pp 667–674

[20] Ritter N, Cooper J (2009). New resolution independent measures of circularity. J Math Imaging Vis.

[21] Ruifrok AC, Johnston DA (2001). Quantification of histochemical staining by color deconvolution. Anal Quant Cytol Histol.

[22] Santanu R, Alok J, Shyam L, Jyoti K (2018). A study about color normalization methods for histopathology images. Micron.

[23] Shen P, Qin W, Yang J, Hu W, Chen S, Li L, Wen T, Gu J: Segmenting multiple overlapping nuclei in h amp;amp;e stained breast cancer histopathology images based on an improved watershed.. In: IET International Conference Biomedical image and signal processing (ICBISP 2015), Beijing, 2015, pp 1–4

[24] Sirinukunwattana K, Raza SEA, Tsang YW, Snead DRJ, Cree IA, Rajpoot NM (2016). Locality sensitive deep learning for detection and classification of nuclei in routine colon cancer histology images. IEEE Trans Med Imaging.

[25] Spanhol FA, Oliveira SLE, Petitjean C, Heutte L Breast cancer histopathological image classification using convolutional neural networks. In: Proc. Int. Conf. Neural networks (IJCNN 2016), Vancouver, 2016

[26] Srivastava N, Hinton GE, Krizhevsky A, Sutskever I, Salakhutdinov R (2014). Dropout: a simple way to prevent neural networks from overfitting. J Mach Learn Res.

[27] Szegedy C, Liu W, Jia Y, Sermanet P, Reed S, Anguelov D, Erhan D, Vanhoucke V, Rabinovich A: Going deeper with convolutions.. In: Proc. IEEE conf. Computer vision and pattern recognition, CVPR, Boston, 2015, pp 1–9

[28] Vincent L (1993). Morphological grayscale reconstruction in image analysis: applications and efficient algorithms. IEEE Trans Image Process.

[29] Xia Y, Zhang B, Coenen F (2016). Face occlusion detection using deep convolutional neural networks. Int J Pattern Recogn Artif Intell.

[30] Yang X, Li H, Zhou X (2006). Nuclei segmentation using marker-controlled watershed, tracking using mean-shift, and Kalman filter in time-lapse microscopy. IEEE Trans Circ Syst.

